# Beyond compliance: evaluating AfriMEDS competencies in South African medical education

**DOI:** 10.1007/s10459-025-10474-z

**Published:** 2025-09-20

**Authors:** Nathaniel Mofolo, Priscilla Mpho Jama, Gina Wisker

**Affiliations:** 1 Department of Family Medicine, School of Medicine, Faculty of Health Sciences, University of Pretoria, Pretoria, South Africa; 2 School of Clinical Medicine, Faculty of Health Sciences, University of the Free State, Bloemfontein, South Africa; 3 School of Management, University of Bath, Bath, UK; 4 Division Student Learning and Development, Faculty of Health Sciences, University of the Free State, Bloemfontein, South Africa; 5 Management Strategy and Organization, University of Bath, Bath, UK

**Keywords:** AfriMEDS, South Africa, Medical curriculum transformation, Competency-based medical education, Assessment, Community-orientated primary care

## Abstract

South African medical education confronts systemic challenges rooted in colonial legacies, demanding transformative pedagogies that redress inequities and integrate community health through culturally responsive frameworks. This convergent mixed-methods study, guided by a transformative paradigm emphasizing ethical engagement and social justice, evaluated the implementation of the AfriMEDS framework, a local adaptation of CanMEDS incorporating community-based education (CBE) and community-oriented primary care (COPC) at the University of the Free State (UFS). Methods included curriculum mapping, document analysis, 15 educator interviews, and surveys of 71 medical interns. Document review revealed that health advocate, leader and manager, and scholar roles were minimally featured in phase guides and that existing assessment tools diverged from CanMEDS recommendations. Educator interviews identified three principal barriers: insufficient faculty development (87% of participants), resource constraints, and misaligned assessment practices. Intern surveys showed only 63% felt leadership training was adequate, 71% felt prepared for CBE, and 72% felt competent in collaboration. These findings expose critical gaps in embedding AfriMEDS competencies, particularly intrinsic roles, within undergraduate training. Our methodological framework highlights how CBE and COPC can serve as catalysts for meaningful curricular reform by fostering sustained collaboration between learners, educators, and communities. We recommend systemic reforms including decolonial pedagogical strategies, robust faculty development in cultural competency, alignment of curricula with national health priorities, and the creation of benchmarked assessment tools that reflect African healthcare contexts and community needs. Failure to implement these reforms risks perpetuating inequity and undermining South Africa’s health transformation agenda.

## Clinical trial number

Not applicable.

## Introduction

Medical education in South Africa stands at critical crossroads where historical injustices converge with contemporary health challenges, demanding a fundamental reimagining of how future healthcare professionals are prepared to serve diverse communities (Slaven, 2017). This imperative extends far beyond merely achieving accreditation compliance, it requires transforming the very foundation of medical training to produce graduates capable of addressing both the persistent inequities born from apartheid and the evolving health needs of a complex, multicultural society. The country’s healthcare system continues to bear the burden of five major health challenges: HIV/AIDS, tuberculosis, non-communicable diseases, interpersonal violence and injuries, and COVID-19-related mental health impacts (Mayosi et al., 2009; Hlongwane & Lowton, 2024). To address these complex realities, South Africa’s medical education system requires transformative approaches that prepare graduates to work effectively in resource-constrained, culturally diverse environments.

### Development of the AfriMEDS framework

Recognizing the limitations of directly applying Global North medical education models to African contexts, the Health Professions Council of South Africa (HPCSA) and the South African Committee of Medical Deans collaborated in 2014 to develop the AfriMEDS competency framework. This adaptation of the Canadian Medical Education Directives for Specialists (CanMEDS) represents an attempt to create culturally responsive medical education standards (De Villiers et al., 2017).

The original CanMEDS framework, developed by the Royal College of Physicians and Surgeons of Canada in 1996, defines seven physician competencies: (1) medical expert, (2) communicator, (3) collaborator, (4) leader, (5) health advocate, (6) scholar, and (7) professional. By 2009, this framework had gained international recognition as a comprehensive approach to competency-based medical education. Dath et al. (2015) demonstrated the evolution from “manager” to “leader” in 2012, reflecting healthcare’s increasing need for reform-focused practitioners. However, the direct application of frameworks developed in the Global North to South African contexts raises fundamental questions about cultural appropriateness, contextual relevance, and the perpetuation of colonial epistemologies within medical education (Wong et al., 2021). These concerns are amplified by evidence that the AfriMEDS curriculum framework, despite its adaptation for African contexts, continues to reflect Western-centric ideologies that may undermine decolonial objectives in medical education (Mnguni, 2024).

### Distinguishing features of AfriMEDS

AfriMEDS differentiates itself from its Canadian predecessor through two essential components: community-based education (CBE) and community-oriented primary care (COPC). These additions reflect Africa’s specific healthcare realities and social determinants of health.

On one hand, CBE represents a shift from traditional hospital-centred training to “decentralized learning” in primary care clinics, district hospitals, and community health centres (Department of Higher Education and Training, 2012; De Villiers et al., 2017; Atwa & Hosny, 2024). For example, medical students participating in CBE might spend weeks in rural clinics where they encounter patients managing chronic diseases without specialist access, learning to provide comprehensive care with limited resources. This approach involves sustained collaboration between students, educators, community members, and healthcare professionals throughout the educational process.

While on the other hand, COPC takes a systematic approach to addressing population health needs through prevention, early detection, and community-based management (American Public Health Association, 1999; Bam et al., 2013). In practice, COPC requires students to conduct community health assessments such as door-to-door surveys, identifying prevalent conditions like HIV, tuberculosis, hypertension, or diabetes, and then design interventions addressing these community-specific health challenges. Both CBE and COPC operate on the understanding that health outcomes are fundamentally shaped by social determinants, requiring interventions that address both individual care needs and broader community contexts.

AfriMEDS emerged ostensibly as a response to South Africa’s persistent healthcare inequalities, which continue reflecting apartheid’s structural legacy despite democratic reforms (World Health Organization, 2013). The framework integrates CBE with COPC approaches, positioning healthcare delivery within community contexts that address diverse linguistic, socio-economic, and cultural realities. A central challenge involves ensuring graduates develop the cultural responsiveness necessary for navigating South Africa’s complex healthcare realities. The answer may lie not in adapted Global North frameworks but in embracing cultural humility paradigms that position healthcare practitioners as perpetual learners rather than cultural “experts” (Tervalon & Murray-García, 1998).

Cultural humility, as conceptualized by Tervalon and Murray-García (1998), emphasizes lifelong self-reflection, power imbalance recognition, and community partnership development, positioning healthcare practitioners as perpetual learners rather than cultural authorities. This approach acknowledges that cultural understanding represents an ongoing process rather than finite knowledge acquisition, particularly relevant for addressing South Africa’s diverse linguistic, socio-economic, and cultural contexts. Educational transformation must therefore integrate decolonial pedagogies that combine indigenous knowledge systems with evidence-based medical practice while addressing systemic healthcare inequities through what Wong et al. (2021) describe as epistemic pluralism (the recognition of multiple valid ways of knowing), cultural safety, and critical consciousness (Reid & Cakwe, 2011).

Implementing AfriMEDS within South Africa’s transition toward national health insurance (NHI) creates additional complexity, requiring interprofessional education approaches that bridge theoretical competencies with diverse, underserved community realities (Republic of South Africa, Department of Health, 2017). Current pedagogical approaches often struggle to achieve this integration, particularly within the collaborator competency domain, where multidisciplinary care coordination becomes essential for addressing the country’s healthcare transformation imperatives.

Critical examination of the CanMEDS-to-AfriMEDS adaptation process reveals significant concerns regarding the suitability of applying Global North competency frameworks to Global South contexts without comprehensive reconceptualization. The adaptation process, while incorporating CBE and COPC components, maintained the Western biomedical paradigm inherent in CanMEDS while merely adding African contextual elements (Mnguni, 2024). This additive methodology exemplifies what Tervalon and Murray-García (1998) critique as the limitations of cultural competence models that assume mastery over cultural knowledge rather than embracing the ongoing, reflexive process of cultural humility. The re-contextualization process inadequately addressed power imbalances embedded within Global North frameworks, potentially perpetuating what Ajani (2024) identifies as “affirmative rather than transformative” approaches that address redistributive justice while neglecting issues of misrecognition and representation. However, its direct application in diverse Global South contexts raised concerns about cultural appropriateness and contextual relevance (Wong et al., 2021).

### Visual framework for implementation evaluation

**Fig. 1:**
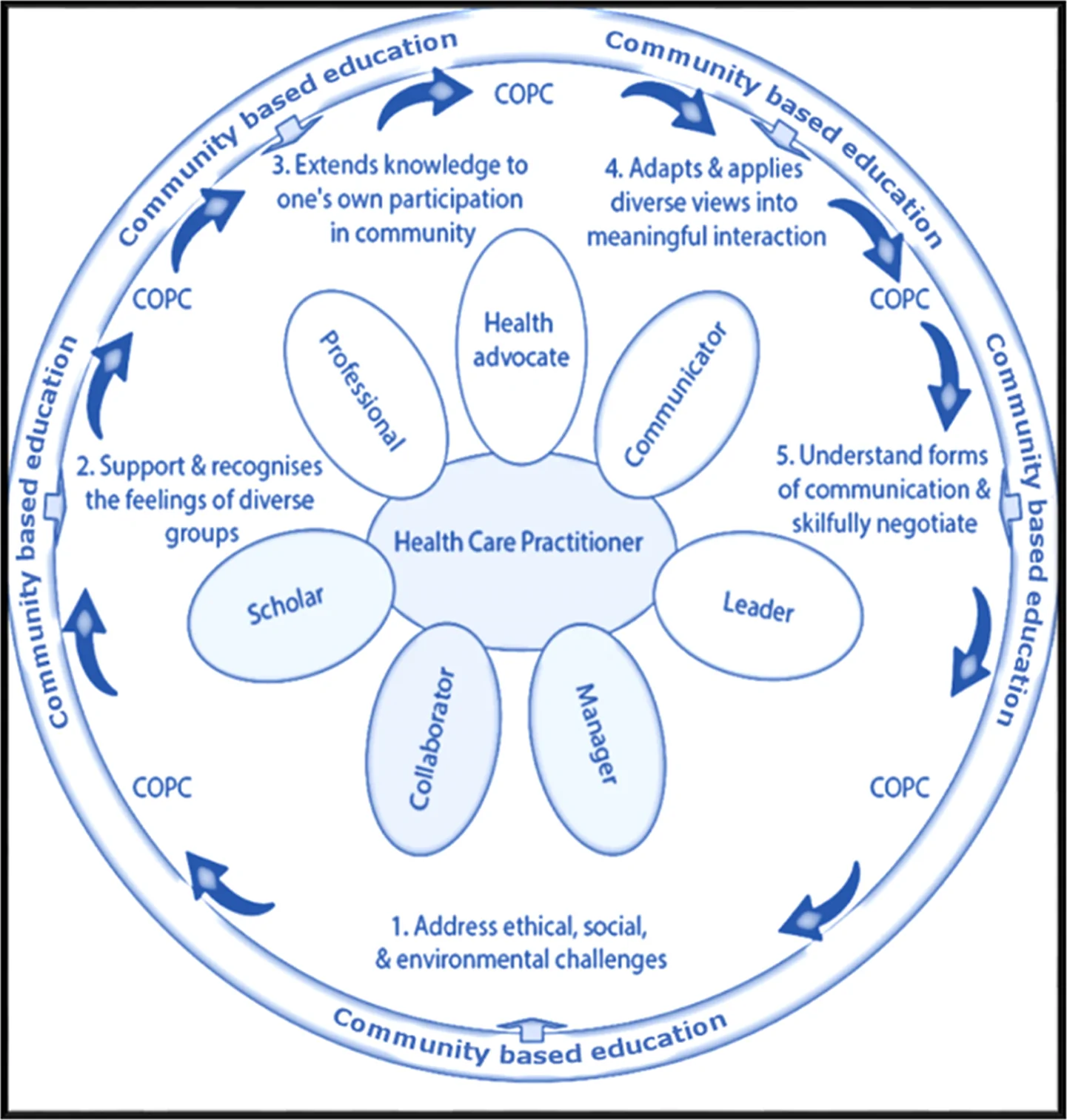
The AfriMEDS framework, adapted from CanMEDS for the South African context. *Copyright © 2015 The Royal College of Physicians and Surgeons of Canada. https://www.royalcollege.ca/en/standards-and-accreditation/canmeds.html. Reproduced with permission

To support systematic evaluation of AfriMEDS implementation, this study developed a visual model (Fig. 1) that synthesizes the framework’s interconnected components. This graphical representation expands upon the original Canadian “flower” configuration by positioning community engagement as the foundational element encompassing all competency domains. The model demonstrates how CBE integrates systematically with COPC principles while maintaining the interconnected nature of competencies essential for African medical practice.

The visual model operationalizes five key COPC principles through a cyclical approach: (1) local health and institutional analysis connects with leader/manager and professional competencies to address ethical, social, and environmental challenges; (2) equity aligns with health advocate and professional roles to support diverse group perspectives; (3) comprehensive care integrates scholar and collaborator competencies to extend knowledge through community participation; (4) practice science social impact engages communicator and scholar roles to adapt diverse viewpoints into meaningful interactions; and (5) service integration around users connects communicator and collaborator competencies to understand communication forms and facilitate skilful negotiation.

This community-centred visual representation serves as an evaluation tool rather than a new foundational framework, providing a practical mechanism for assessing how well medical schools integrate African-contextualized competency training with community health priorities.

### UFS as case study

The University of the Free State (UFS) medical school in Bloemfontein provides a compelling case study for examining these implementation challenges, representing the only South African institution offering a five-year undergraduate medical program within the contemporary healthcare transformation landscape characterized by resource constraints and accelerated curriculum demands. Historically, UFS enforced policies of exclusion and parallel-language instruction that segmented students and perpetuated race-based inequity (Suransky & Van der Merwe, 2016). Despite post-1994 reforms that improved access and shifted to English as the medium of instruction from 2016, faculty and students continue to grapple with the legacies of segregated clinical training, resource scarcity, and inequitable language policies. Findings from UFS case study reveal that, although alignment with AfriMEDS is nominally present in curriculum documents, actual implementation remains inconsistent. The implementation challenges at UFS are not isolated; they mirror similar struggles in medical education reform across the Global South.

### International transferability

Comparable efforts in other Global South regions underscore shared challenges and the promise of south–south collaboration. In sub-Saharan Africa, Makerere University and the University of Ibadan have implemented CBE-infused competency frameworks despite resource constraints (McKenzie-White et al., 2022). Latin American medical schools, notably in Brazil and Colombia, have confronted analogous obstacles in tailoring competency-based assessment strategies to local health priorities and cultural contexts (Armijo-Rivera et al., 2025). In India, the National Medical Commission’s 2019 mandate for CBE across medical colleges disclosed faculty resistance, infrastructural limitations, and the need for extensive capacity-building to support the shift from time-based to competency-based curricula (Gopalakrishnan et al., 2022). These parallel experiences not only illuminate universal barriers to competency-based medical education in resource-constrained settings but also highlight opportunities for mutual learning and adaptation across the Global South.

The South African case study offers significant international transferability value for medical education systems grappling with similar colonial legacies, resource constraints, and health workforce distribution challenges characteristic of many Global South contexts. The experience of implementing competency-based frameworks originally designed for resource-rich, monocultural contexts within complex, multicultural, resource-constrained environments, provides crucial insights for medical education transformation efforts across sub-Saharan Africa, Latin America, and South Asia. The methodological approaches employed in this study, particularly the transformative research paradigm’s emphasis on stakeholder co-creation and power dynamic examination, offer replicable frameworks for investigating educational reform intersections with social transformation agendas in diverse international contexts.

### Research objectives

This study employs a transformative research paradigm to address the power dynamics and cultural complexities inherent in medical education reform, recognizing how traditional hierarchical structures within medical education may impede curriculum transformation by marginalizing student and community perspectives while privileging established academic authority (Statistics South Africa, 2013). The investigation examines how AfriMEDS implementation can be evaluated and enhanced through stakeholder co-creation processes that authentically serve community health priorities while meeting accreditation standards. By positioning stakeholders as knowledge co-creators rather than research subjects, this approach challenges the reproductive rather than transformative mode that continues characterizing South African medical education despite policy imperatives for transformation (Reid, n.d.).

This research, therefore, approaches AfriMEDS implementation not as a technical exercise, but as one deeply embedded in the politics of social accountability, cultural humility, and stakeholder co-creation (Tervalon & Murray-García, 1998; Mnguni, 2024).

In summary, the UFS case demonstrates that meaningful transformation in South African medical education requires not only adopting international best practices but also critically addressing enduring structural, cultural, and epistemic inequities. The adapted AfriMEDS model, when robustly contextualized, holds the potential to catalyse curricular reform that is both socially accountable and responsive to South Africa’s evolving health landscape.

## Methods

### Research design and methodology

This study employed a convergent parallel mixed-methods design situated within a transformative research paradigm, which foregrounds ethical engagement, social justice, and the redress of power imbalances throughout the research process to evaluate AfriMEDS implementation at the UFS. The transformative paradigm emphasizes participatory, relationship-building approaches in which stakeholders particularly those from marginalized communities contribute as co-creators of knowledge, thereby aligning scholarly inquiry with societal needs and advancing social change goals (Mertens, 2005). Consequently, the transformative paradigm was selected for its explicit interrogation of cultural complexity and structural inequities and for positioning participants as knowledge co-constructors, which facilitated simultaneous examination of individual competency development and systemic barriers to medical education transformation in post-apartheid South Africa (Mertens, 2007).

### Philosophical foundations of the transformative paradigm

The transformative paradigm guiding this study adopts a multi-faceted approach to social justice and equity within medical education research. This framework foregrounds collaborative knowledge creation by engaging medical interns, educators, and community stakeholders as co-creators whose lived experiences and local priorities inform both inquiry and action (Mertens, 2007). How does this approach address the complexities of post‐apartheid South African medical education?

The paradigm recognizes that multiple, contextually situated realities coexist within this educational landscape, necessitating methodological flexibility and systematic triangulation. Rather than adhering to positivist hierarchies, the framework privileges collaborative, praxis-oriented epistemologies that evaluate evidence according to its potential to catalyse structural and curricular transformation. This orientation mandates a mixed‐methods design that integrates document review, interviews, and surveys to holistically capture the complex, culturally inflected processes through which the AfriMEDS framework has been implemented and assessed at the UFS (Creswell & Plano Clark, 2017).

### Study setting and contextual considerations

UFS, the only South African institution offering a five-year undergraduate medical programme, provided a unique context for investigating AfriMEDS implementation. Transition from a dual-language policy (Afrikaans and English) from 1993 to English-only instruction in 2016 (while still supporting Afrikaans, Sesotho, and isiZulu) in tutorials, illustrates the institutional shifts influencing curriculum delivery.

### Data collection methods

Three complementary data collection methods were employed: document analysis, semi-structured educator interviews, and an online intern survey. This triangulated approach enabled comprehensive examination of AfriMEDS implementation from multiple stakeholder perspectives while addressing potential biases inherent in single-method studies.

#### Document analysis

Six key institutional documents were systematically reviewed: the 2017 HPCSA accreditation report, the three phase guides (Phases I–III), the undergraduate rule book, and the 2019 MBChB curriculum review summit. Analysis focused on competency integration frequency, alignment of assessment methods, and congruence between policy and practice.

#### Semi-structured interviews

Fifteen educators from diverse clinical disciplines participated in 45–60-minute interviews via online Blackboard Collaborate. The guide explored: (a) assessment methods for each competency, (b) implementation challenges, (c) gaps in tools, (d) best practices, and (e) management support needs.

#### Online survey

An online questionnaire was distributed to 225 first- and second-year interns doing internship training in 2020; 71 responded (31.6%). Sections addressed demographics, overall internship experience, self-assessed preparedness across eight AfriMEDS domains (5-point Likert scales, dichotomized for accreditation relevance), and open-ended questions on training gaps.

### Data analysis procedures

Data analysis was conducted by the principal researcher with verification by the co-investigator to ensure reliability. Qualitative data analysis involved independent coding by both researchers, with discrepancies resolved through discussion and consensus. To mitigate social desirability bias stemming from the principal investigator’s institutional role, all interviews were conducted by an independent researcher not affiliated with the project.

#### Quantitative analysis

Quantitative analysis employed descriptive statistics, calculated using STATA version 12, to examine competency assessment perceptions among medical interns who graduated from the institutions’ undergraduate study program. To get the clearest possible picture of intern readiness, we grouped their responses into two simple categories: ‘prepared’ and ‘not prepared ‘. This was done in order to align with the HPCSA accreditation requirements, which mandate clear pass-fail determinations for graduate competencies, interns’ 5-point Likert responses (strongly disagree–strongly agree) were dichotomized into “agree (prepared)” (strongly agree/agree) versus “not agree (not prepared)” (neutral/disagree/strongly disagree). This binary classification parallels high-stakes clinical skill assessments and provides curriculum planners with unequivocal metrics for gauging readiness across the eight AfriMEDS domains. Composite scores expressed as the percentage of “agree” responses per competency were then categorized into three performance bands (0–49%: insufficient; 50–79%: moderate; 80–100%: adequate) to facilitate targeted remediation and resource allocation (Frank et al., 2010; Ten Cate & Scheele, 2007). Although collapsing ordinal data sacrifices nuance between “agree” and “strongly agree,” this methodological compromise prioritizes regulatory alignment and operational clarity over analytical granularity, ensuring that only interns meeting or exceeding expected proficiency thresholds are deemed competent for unsupervised practice (Mertens, 2010).

#### Qualitative analysis

Interview transcripts and open-ended survey responses underwent thematic analysis following Braun and Clarke’s (2006) six-phase approach. Initial codes were generated inductively, then organized into themes representing patterns across the dataset. A joint display was constructed to integrate quantitative satisfaction ratings with qualitative themes, enabling identification of convergent, divergent, and complementary findings across data sources.

### Participant protection and informed consent

All participants provided written informed consent prior to participation, with consent processes tailored to address the specific risks and benefits associated with each stakeholder group. Educators received detailed information about interview procedures, data handling protocols, and their rights to withdraw from the study without consequence. Medical interns were informed about survey confidentiality measures and their right to skip questions or withdraw at any point during completion.

#### Vulnerable population considerations

Special attention was paid to potential power dynamics affecting medical interns, who might feel obligated to participate or provide socially desirable responses given their junior position in the medical hierarchy. Clear statements were provided that participation was voluntary, would not affect their internship evaluations, and that individual responses would remain confidential. No identifiable information was collected beyond basic demographic data necessary for analysis.

#### Cultural sensitivity

Data collection protocols acknowledged participants’ diverse cultural backgrounds and language preferences, consistent with UFS’s multilingual educational environment. While interviews and surveys were conducted in English, participants were encouraged to use terminology or expressions from their preferred languages when necessary to convey meaning accurately. This approach recognized that imposing monolingual data collection might disadvantage participants whose first language was not English.

### Data protection and confidentiality

Robust data protection measures were implemented throughout all phases of the research to safeguard participant confidentiality and institutional sensitivity. Individual online interviews were conducted via Blackboard Collaborate under the UFS’s institutional license, ensuring compliance with university data governance policies. Audio recordings were downloaded and transcribed using Otter.ai artificial intelligence transcription software, accessed through the university’s institutional licensing agreement that provided enhanced security protections for sensitive research data.

#### AI transcription ethics

Participants were explicitly informed that AI transcription software would process their recorded responses. This disclosure explained data transfer protocols, potential risks associated with cloud-based processing, and verification procedures employed to ensure transcription accuracy. All AI-generated transcripts underwent comprehensive human review and verification, with the researcher listening to the original audio recordings and making necessary corrections to ensure accuracy and maintain participant voice integrity.

#### Data storage and retention

All research data were stored on password-protected, encrypted institutional servers with access limited to the research team. Physical documents were secured in locked filing cabinets within restricted-access university offices. Data retention policies followed both UFS and University of Bath requirements, with personal identifiers to be destroyed five years post-publication while anonymized datasets retained for potential secondary analysis or replication studies.

### Institutional and community benefit

The research provided a direct benefit to participating institutions and the broader South African medical education community. Findings were shared with UFS leadership during the research forums, to inform curriculum development decisions, and recommendations were developed to address specific implementation challenges identified during data collection. This approach aligned with transformative research principles by ensuring that knowledge generation served community transformation goals rather than purely academic interests.

#### Reciprocity and knowledge sharing

Results are being disseminated through multiple channels to maximize impact on medical education and practice. The dissemination plan includes; academic publications complemented by institutional reports, professional conference presentations, and policy briefs targeting medical education regulators. This multi-modal strategy ensures that findings extend beyond academic circles and inform both practice and policy development.

### Reflexivity and researcher positionality

This study operationalized critical reflexive praxis (CRP), a systematic approach involving self-reflection on researcher positionality throughout the research process to examine how institutional positioning and underlying assumptions shaped study design, data collection, and interpretation (Sims & Saunders, 2025). CRP challenged entrenched hierarchies in medical education research that privilege dominant biomedical perspectives over marginalized voices (Alexander et al., 2020; Mertens, 2007). The principal investigator’s dual role as medical practitioner and UFS academic, provided nuanced participant access while introducing potential unconscious bias. To address this insider-outsider position, interviews were conducted by external researchers, qualitative analysis was independently verified by non-UFS co-investigators, and findings were triangulated across multiple sources. The research team’s multi-lingual capabilities enhanced interpretation of participant perspectives, particularly where code-switching emerged. CRP principles shaped concrete actions including expanding intern participation, integrating community stakeholder viewpoints, and deliberately foregrounding community partnership as alternatives to traditional expert-led models (Mertens, 2007).

### Data integration and validation

The convergent parallel mixed-methods design facilitated comprehensive data integration through systematic triangulation across multiple stakeholder groups and data sources (Creswell & Plano Clark, 2017). Qualitative data achieved theoretical saturation through thematic analysis using NVivo 12 software. Quantitative analysis generated positive composite core competency scales using 5-point Likert measurements, with Cronbach’s alpha reliability coefficients ranging from 0.78 to 0.90 across competency domains. Quantitative ratings of interns’ competency preparedness were systematically compared with themes from qualitative educator interviews and document analysis of phase guides. Member checking involved sharing synthesized results with selected interns and faculty to verify that interpretations accurately reflected their experiences and to identify any overlooked nuances. This process of cross-validation, grounded in consistent use of the same competency questions across methods, strengthened internal validity and credibility, while adherence to institutional ethics and data-management policies upheld trustworthiness and confirmability.

### Study limitations and trustworthiness

This evaluation demonstrates several methodological limitations that constrain generalizability while systematically applying trustworthiness criteria to enhance confidence in findings. The single-institution case study design limits direct transferability to other medical schools, though detailed contextual description enables readers to assess applicability to their institutional settings, a consideration particularly relevant for resource-constrained environments implementing competency-based medical education frameworks.

The 31.6% response rate for the intern survey, while acceptable for online medical education research, raises questions about potential selection bias affecting quantitative findings. Moreover, the decision to dichotomize 5-point Likert scale data and its limitation have been acknowledged in the methods section under quantitative analysis.

Future research could benefit from analysing full-scale data to capture nuanced response patterns while implementing strategies to enhance participant response rates across diverse institutional contexts implementing similar transformative educational frameworks.

#### COVID-19 impact

Data collection during the COVID-19 pandemic affected both intern availability and program delivery, potentially influencing participant responses about clinical exposure and assessment experiences. This temporal context was systematically documented and considered during interpretation to distinguish pandemic-specific challenges from systemic implementation issues.

## Findings

The convergent parallel mixed-methods design yielded comprehensive insights into AfriMEDS competency implementation and assessment across multiple stakeholder perspectives at the UFS. This transformative paradigm investigation reveals both systemic challenges and opportunities for curriculum reform that extend beyond technical implementation issues to encompass deeper questions of educational equity and community responsiveness in South African medical education.

### Document analysis: institutional framework and curriculum alignment

The systematic analysis of six institutional documents, comprising the 2017 HPCSA accreditation report, the three phase guides (Phases I–III), the undergraduate rule book, and the 2019 MBChB curriculum review summit, reveals significant disparities in competency emphasis across the five-year curriculum structure. These documents represent the formal institutional commitment to AfriMEDS implementation, yet their analysis exposes fundamental misalignments between policy intention and curricular reality.

The 2017 HPCSA accreditation report flagged nine systemic deficiencies at UFS: (1) absence of Sesotho language training for patient communication; (2) limited interprofessional learning; (3) informal curriculum-review feedback loops; (4) inadequate CBE exposure; (5) inequitable student-selection processes; (6) staff shortages amid rising enrolments; (7) inconsistent assessment practices; (8) short clinical rotations with no continuity of care; and (9) the absence of longitudinal integrated training sites. Collectively, these gaps underscored misalignment between AfriMEDS goals and actual programme delivery and provided a regulatory benchmark for the present study’s critique of implementation quality.

The quantitative frequency analysis of phase guides demonstrates pronounced variations in competency representation across program phases, with implications for students’ progressive competency development. Professional competencies appear most frequently in Phase I (*n* = 15), declining substantially in subsequent phases (Phase II: *n* = 7; Phase III: *n* = 1), while CBE receives concentrated attention in Phase II (*n* = 32) but minimal emphasis elsewhere. This uneven distribution suggests a curriculum structure that may inadequately support the systematic, longitudinal development of competencies that characterizes effective competency-based medical education (Frank et al., 2010).

Document analysis demonstrated that health advocate competencies were absent from Phase I guides, and with only a single mention of health advocate recorded in Phase III documentation. While leader/manager competencies appeared only five times in Phase I and were absent from Phase II entirely. This absence contrasts with South Africa’s healthcare transformation agenda and the explicit community-oriented focus of the AfriMEDS framework. The HPCSA’s adaptation of CanMEDS specifically emphasized CBE and COPC to address persistent healthcare inequities (HPCSA, 2014). However, institutional documents demonstrated limited integration of advocacy skills development, a pattern that appeared consistently across subsequent stakeholder perspectives.

The assessment method documentation reveals equally concerning misalignments between stated competency goals and evaluation practices. While the institutional framework articulates a commitment to holistic competency development, the assessment tools described in phase guides predominantly reflect traditional biomedical evaluation approaches rather than the multi-dimensional assessment strategies recommended for competency-based frameworks (Bandiera et al., 2006). This disconnect between aspirational policy and operational reality exemplifies what scholars have identified as the persistent challenge of translating competency frameworks into meaningful educational practice (Gaboury et al., 2018).

### Educator perspectives: implementation challenges and professional insights

The semi-structured interviews with 15 educators across all program phases illuminate the complex realities of AfriMEDS implementation from those directly responsible for curriculum delivery. These perspectives, gathered through semi-structured interviews conducted by an external researcher to minimize hierarchical bias, reveal both a professional commitment to CBE and significant institutional rigidity to effective implementation.

### Competency-specific implementation patterns

Educators demonstrated strong consensus regarding medical expert competencies, with 12 out of 15 (80%) affirming adequate implementation. This finding aligns with existing literature suggesting that clinical competencies, being more concrete and measurable, receive more consistent attention in medical curricula (Whitehead et al., 2015). However, educator confidence declined markedly when discussing intrinsic competencies, those requiring complex social and contextual skills development.

The leader and manager competency received the lowest educator endorsement (eight out of 15 educators, 53%), with participants articulating profound uncertainty about pedagogical approaches and assessment methodologies. One educator observed:

We expect students to demonstrate leadership during clinical rotations, but we’ve never explicitly taught them what medical leadership means in the South African context. How do you assess someone’s ability to manage resources when they’ve never been given the opportunity to make those decisions?

This quotation encapsulates the fundamental challenge identified across multiple competency domains: the expectation of student performance without corresponding educational scaffolding or authentic assessment opportunities.

Professional competencies, while receiving universal educator acknowledgment (100%), established implementation approaches that relied heavily on implicit learning through clinical modelling rather than structured competency development. Educators described professionalism as “*caught rather than taught*,” revealing a reliance on implicit socialization rather than structured pedagogical strategies. This gap highlights an urgent need for formal faculty development in teaching and assessing professional competencies, as the current approach may inadequately prepare students for the complex ethical challenges inherent in the South African healthcare context. The reliance on tacit professional socialization becomes problematic when considering the diverse cultural backgrounds of students and the need for explicit discussions about professional identity in post-apartheid healthcare settings.

### Systemic barriers to implementation

Educators identified five primary structural barriers that consistently impeded effective AfriMEDS implementation: (1) inadequate faculty development, (2) insufficient time allocation, (3) limited clinical exposure opportunities, (4) resource constraints, and (5) the persistent impact of COVID-19 disruptions. These barriers operate synergistically, creating what participants described as a “perfect storm” of implementation challenges.

Faculty development emerged as a critical bottleneck, with educators acknowledging their own uncertainty about competency-based assessment methodologies. One senior educator reflected:

I’ve been teaching medicine for twenty years, but this competency framework requires a completely different approach. I’m comfortable assessing clinical skills, but how do I fairly evaluate a student’s advocacy potential or their scholarly mindset? We need training, but there’s no time for professional development when we’re already struggling to cover the essential clinical content.

This perspective reflects broader challenges in competency-based medical education implementation globally, where faculty capacity building represents a persistent implementation barrier (Carraccio et al., 2002). However, the South African context adds additional complexity through resource constraints and the competing demands of healthcare service delivery in under-resourced settings.

Time pressures were universally cited across educator interviews, with particular emphasis on the compressed nature of UFS’s five-year program structure. Unlike other South African medical schools offering six-year programs, UFS educators described constant pressure to balance competency development with content coverage. This temporal constraint demonstrates to particularly impact the development of reflective competencies such as scholarship and advocacy, which require sustained and iterative opportunities for development to engage.

### Assessment methodology gaps

The qualitative data obtained through semi-structured educator interviews illuminated significant inconsistencies and limitations within current assessment practices for non-medical expert competencies in the AfriMEDS-aligned curriculum. Although educators collectively affirmed the theoretical intent to evaluate all eight AfriMEDS domains, comprising the seven physician roles and community-based education, the absence of standardized, validated tools resulted in considerable variability, particularly for intrinsic competencies such as leadership, collaborator, and CBE.

A pervasive theme was the reliance on informal or impressionistic evaluation techniques in lieu of structured, competency-based tools. Across multiple domains, participants described an environment in which formal assessment mechanisms were largely absent. This finding was typified by an educator’s observation:

Students arrive in communities without understanding the social determinants of health or how to communicate across cultural and linguistic differences. We assess their clinical performance, but miss the deeper competencies around community understanding and advocacy that are supposed to define the AfriMEDS approach.

Educators, further, cited logistical and contextual obstacles impeding robust assessment. These included inherent language barriers within the diverse South African medical context, a scarcity of CBE placements that facilitate meaningful community engagement, and a lack of faculty preparation for evaluating competencies beyond the clinical domain. The absence of structured observation rubrics and a standardized portfolio system reinforced an assessment culture that privileged biomedical knowledge and clinical proficiency at the expense of holistic, community-responsive practice.

In summary, the assessment infrastructure did not align with either the explicit objectives of the AfriMEDS framework or the evolving accreditation criteria set by the HPCSA (cf. De Villiers et al., 2017). This methodological gap not only undermined authentic competency verification but risked perpetuating superficial compliance with national and institutional priorities.

### Best practices in assessing AfriMEDS competency roles

UFS School of Medicine educators highlighted three key strategies to strengthen AfriMEDS assessment. First, analytic rubrics based on the Calgary-Cambridge guide both formative OSCEs and peer-assessed presentations, ensuring clarity and consistency. Second, authentic workplace assessments embed students in interprofessional teams during rural Trompsburg (CBE and COPC site) rotations, offering real‐world feedback on collaboration, leadership, and cultural responsiveness. Third, secure digital platforms (Blackboard, Question mark) and external examiners uphold fairness and validity under UFS assessment policies. Several of these approaches are summarized in the recommendations.

### Medical intern perspectives: preparedness for professional practice

The online survey of 71 medical interns completing first and second-year internships provides crucial insights into the effectiveness of AfriMEDS implementation from the perspective of recent graduates transitioning into professional practice. With a response rate of 31.6%, these perspectives offer valuable feedback on competency development outcomes and perceived preparedness for clinical responsibilities.

### Overall preparedness and competency confidence

Medical interns reported high levels of preparedness for internship responsibilities, with 80% expressing confidence in their overall readiness for clinical practice. This positive assessment aligns with the strong performance in medical expert competencies, where 91% of interns felt adequately prepared for clinical diagnosis and management responsibilities. However, this confidence was not uniformly distributed across all competency domains.

The quantitative assessment established a clear hierarchy of perceived preparedness, with biomedical competencies receiving the highest confidence ratings and socially oriented competencies demonstrating lower satisfaction scores. Professional competencies achieved 84% satisfaction, while scholar competencies reached 90%, suggesting that certain non-clinical competencies received adequate attention during undergraduate training.

### Critical competency gaps

Triangulated survey data from 71 medical interns yielded complementary insights, with quantitative and qualitative strands exposing critical, domain-specific deficits. Medical interns demonstrated high competency ratings (> 80%) for medical expert roles but significant gaps emerged in non-clinical competencies: leadership and management (63%), CBE (71%), collaborator (72%), health advocate (78%), and communicator (79%).

Open-ended responses illuminated the lived reality beneath these figures. One intern captured the theoretical-practical disjuncture, stating:

I can diagnose and treat patients effectively, but I struggle to understand why the same conditions keep recurring in certain communities. We learned about social determinants theoretically, but I don’t feel equipped to address them practically or advocate for community-level interventions.

This sentiment was broadly echoed in relation to other non-clinical competencies. With respect to interprofessional collaborator, interns described feeling insufficiently prepared for the complex, often conflictual, team dynamics characteristic of South African public health facilities. Specific deficits were highlighted around conflict resolution and navigating ambiguities in role delineation. Although they could articulate the importance of teamwork as a principle, many lacked practical experience applying these skills in high-pressure, resource-constrained environments.

The results pertaining to CBE offered a stark example of the distinction between theoretical satisfaction and substantive readiness. Despite a 71% positive rating, qualitative evidence suggested that CBE was primarily experienced as a tick-box exercise, limited in duration, focused on clinical tasks, and rarely demanding sustained engagement with community members or collective health advocacy efforts.

Finally, the leader and manager domains surfaced as acute vulnerabilities. A significant proportion of interns reported inadequate exposure to formal leadership responsibilities during undergraduate training, describing scenarios in which they were thrust into authority roles without adequate scaffolding or mentorship:

When suddenly expected to lead a ward team, I realized I had only ever seen these roles performed but had never practiced them myself.

Collectively, these findings expose not isolated anomalies, but rather, systemic weaknesses in nurturing and assessing the full spectrum of AfriMEDS core competencies, a shortfall that runs the risk of undermining the very objectives driving the nation’s curriculum reform agenda (Mnguni, 2024; Whitehead et al., 2015).

### Language and cultural competency challenges

A recurring theme in intern feedback concerned language barriers and cultural competency challenges. Despite South Africa’s multi-lingual healthcare environment, interns reported limited preparation for cross-cultural communication and community engagement. This finding assumes particular significance given the AfriMEDS framework’s explicit emphasis on community-oriented care and cultural responsiveness.

The language competency gap reflects broader challenges in South African medical education, where instruction occurs primarily in English while patient populations may communicate in any of the 11 official languages. Interns described situations where communication barriers impeded both clinical care delivery and community engagement opportunities:

I completed my community rotation, but I couldn’t communicate effectively with many patients in their home language. This limited my ability to understand their perspectives on health and illness, which I think is supposed to be central to the community-based education experience.

This feedback suggests that implementing CBE may not adequately address the linguistic and cultural competencies necessary for effective COPC.

### Convergent analysis: integrating stakeholder perspectives

The triangulation of document analysis, educator interviews, and intern surveys reveals consistent patterns across data sources that illuminate both systemic challenges and potential intervention points for AfriMEDS implementation improvement. This convergent analysis demonstrates the value of transformative paradigm inquiry in exposing structural inequities while identifying opportunities for meaningful educational reform.

### Consistent implementation gaps

Three competency domains emerged as consistently under implemented across all data sources: leader and manager, health advocate, and CBE. These competencies share common characteristics as socially oriented, contextually-dependent capabilities that require sustained development opportunities and authentic assessment environments. Their consistent under representation directly contradicts the visual model’s principle (Fig. 1) of connecting local health analysis with the leader and manager competency, health advocate, and CBE.

The alignment between educator uncertainty and student unpreparedness in these domains indicates that competency gaps compound throughout the educational continuum. When educators lack confidence in teaching and assessing particular competencies, students receive inadequate preparation, resulting in graduates who enter practice without essential professional capabilities. This pattern perpetuates itself as these inadequately prepared graduates may eventually become educators themselves, lacking the competency-specific knowledge necessary for effective teaching.

### Assessment methodology limitations

All stakeholder groups identified the assessment methodology as a critical implementation barrier, though from different perspectives. Documents uncovered a misalignment between assessment tools and competency requirements; educators described uncertainty about assessment approaches, and interns reported inconsistent evaluation experiences. This convergence suggests that assessment represents a fundamental implementation challenge requiring systematic intervention.

The repeated identification of “no formal assessment” across multiple competency domains indicates an implementation approach that may rely too heavily on implicit evaluation rather than explicit competency verification. This approach becomes problematic for intrinsic competencies that require structured development and systematic assessment to ensure acquisition.

### Contextual implementation barriers

The South African healthcare context creates unique implementation challenges that emerged consistently across stakeholder perspectives. Resource constraints, linguistic diversity, cultural complexity, and historical inequities in healthcare access all influence AfriMEDS implementation in ways that may not be adequately addressed in the original CanMEDS framework. These contextual factors require explicit consideration in curriculum design and implementation strategies.

The COVID-19 pandemic emerged as a significant implementation disruptor, particularly affecting CBE opportunities and collaborative learning experiences. However, stakeholder responses suggested that pandemic-related challenges may have exposed pre-existing implementation weaknesses rather than creating entirely new problems.

Here are quotes from the educators regarding COVID-19’s impact on medical education:

From the interviews with educators, one participant stated:

What happened now with the COVID, is that there were extreme pressures with the clinical settings for the students’ doing deliveries, that is, on the ability to have that chance to deliver without the risk. Because at that stage the fourth-year students work under the direct supervision of a midwife, and we had large numbers of the midwives that were affected. The supervision was adversely impacted. What we did in that period, they were limited, there was a shift to more online, sort of, training, and giving them sort of case scenarios which they needed to work through.

Educators reflected:

Because normally, we say a student has two textbooks. One is written textbook, the other one is a patient. so, at the moment under COVID is a limit to that percentage of 50:50 textbook, both patient and written stuff is going more in the favour of the written part. At the moment, we’re using more of the fourth industrial revolution instruments, like the simulation lab, where we listen to the heart sounds. And then, we practice the resuscitation on the mannequins.

and even now the exams that we do, the so called OSCE exams, a lot of them are not live like we used to in the past,

I missed out that human contact with the students, this year. Exposure to patients was diminished this year and they could see it, having an effect on the clinical skills.

### Implications for transformative curriculum reform

These findings, viewed through the lens of transformative paradigm inquiry, suggest that effective AfriMEDS implementation requires more than technical curriculum adjustments. The consistent under representation of socially oriented competencies and the persistent challenges in community engagement reflect deeper questions about the purpose and orientation of medical education in post-apartheid South Africa.

The evidence suggests a need for curriculum reform that explicitly addresses power dynamics in healthcare, centers community voices in educational planning, and develops authentic assessment methodologies that reflect the complex realities of South African healthcare practice. Such reform would require an institutional commitment to transformative change rather than superficial compliance with accreditation requirements.

The transformative paradigm lens reveals that current implementation challenges may reflect broader tensions between maintaining traditional biomedical education approaches and genuinely embracing community-oriented, socially responsive medical education. Resolution of these tensions requires an institutional commitment to meaningful transformation rather than cosmetic curriculum modifications.

These findings provide a foundation for understanding both the challenges and opportunities inherent in implementing CBE in complex, resource-constrained environments. The convergent evidence from multiple stakeholder perspectives shows the need for systematic, contextually responsive approaches to curriculum reform that address both technical implementation barriers and deeper questions of educational purpose and social responsibility in medical training.

## Discussion

### Systemic barriers to AfriMEDS implementation

The persistence of misalignment between AfriMEDS’ intended outcomes and UFS’s assessment practices can be traced to four entrenched, interrelated systemic barriers common in CBE implementation internationally (Caverzagie et al., 2017). First, regulatory misalignment undermines coherent progression: multiple accrediting bodies (HPCSA, university structures, department curricula) operate with overlapping yet distinct mandates, impeding the seamless integration of AfriMEDS into teaching, assessment, and promotion policies. Second, the educational-clinical divide perpetuates silos: medical educators design curricula in isolation from frontline clinicians, while service demands constrain teaching time, yielding assessments that prioritize easily measured knowledge over complex professional roles.

Third, the absence of clearly defined, measurable outcomes for non-expert roles (leader, advocate, collaborator) leaves faculty without shared benchmarks, so assessment defaults to conventional tests ill-suited for these competencies (Norcini, 2023). Finally, a culture lacking mutual accountability allows superficial compliance brief workplace observations or token community rotations without genuine curricular transformation. How might these barriers be addressed? Overcoming these challenges requires harmonizing regulatory requirements across all stakeholder levels, co-designing curriculum and care delivery with clinical partners, establishing explicit, measurable milestones for each competency domain, and building shared accountability through transparent reporting and interprofessional governance structures.

### Assessment misalignment and competency gaps

Document analysis evidenced systematic assessment misalignment, with traditional evaluation methods poorly suited to competency-based education requirements. The predominance of multiple-choice questions and conventional examinations for assessing complex competencies like health advocacy and leadership represents fundamental paradigmatic mismatch. These findings support critical perspectives questioning whether imported frameworks adequately address local contextual needs (Mnguni, 2024).

Intern survey responses illuminated significant competency gaps, particularly in non-medical expert roles. Our survey shows that just 63% of interns feel adequately trained in leadership roles, 71% in CBE, and 72% in collaborative practice. These patterns suggest that while medical schools successfully develop clinical expertise, they struggle to cultivate broader professional competencies essential for healthcare transformation. The emphasis on technical medical knowledge at the expense of leadership, advocacy, and community engagement capabilities potentially undermines graduates’ capacity to drive health system reform.

Cultural humility and community engagement represent particularly challenging areas for assessment and development. Traditional academic environments often privilege theoretical knowledge over contextual understanding and community partnership skills. The persistent dominance of Western-centric pedagogical approaches, despite AfriMEDS’s Afrocentric aspirations, may contribute to these deficiencies. This finding reinforces arguments for decolonial pedagogical approaches that integrate indigenous knowledge systems with evidence-based medical practice (Tervalon & Murray-García, 1998).

### Transformative paradigm effectiveness

The transformative research paradigm proved effective in exposing hidden structural inequities within medical education systems. By positioning stakeholders as knowledge co-creators rather than research subjects, this approach illuminated power dynamics that traditional evaluation methods might obscure. Educator interviews highlighted how hierarchical structures within medical education impede authentic curriculum transformation by marginalising student and community perspectives while privileging established academic authority.

CBE emerged as particularly crucial for sustainable reform, though current implementation remains inadequate. The transformative paradigm’s emphasis on authentic community partnership revealed significant gaps between policy intentions and practical reality. Educators reported minimal meaningful community integration, with CBE often reduced to brief rotations rather than sustained partnership development. This finding underscores the need for structural reforms that prioritise genuine community engagement over superficial compliance with accreditation requirements.

The paradigm’s effectiveness extended to revealing assessment inadequacies that traditional evaluation approaches might normalise. By examining curriculum implementation through transformative lenses, the study identified how current assessment practices may perpetuate rather than challenge existing inequities in medical education and healthcare delivery.

### Decolonial implications and cultural contextualisation

In South Africa’s post-2016 shift to an English-only medium of instruction at UFS, even while nominally retaining Afrikaans, Sesotho, and isiZulu in tutorial settings, the university’s historical dual-language policy (1993–2016) laid groundwork that neither fully prepared interns for exclusive English immersion nor systematically developed their home-language competencies. Under the parallel-medium regime (1993–2016), students could learn and be assessed in Afrikaans or English, but this model perpetuated siloed instruction and limited cross-language practice. The 2016 policy pivot to English-only lectures increased curricular pressure and narrowed everyday use of Sesotho and isiZulu to optional tutorials, reducing sustained exposure and formal assessment in these languages. Consequently, interns reported communication gaps not simply as individual deficits but as a direct product of policy: they lacked sufficient structured instruction in Sesotho, the home language of 72.2% of the Free State population in which they were trained, and in isiZulu, the most widely spoken home language nationally at 24.4% and therefore prevalent across the provincial services to which interns are subsequently allocated (Statistics South Africa, 2025), were offered only sporadic “language support” sessions, and received no credit for multi-lingual patient interviews. This policy-practice disjunction has thus entrenched English dominance while marginalizing indigenous languages, exacerbating interns’ inability to establish therapeutic relationships in patients’ mother tongues and undermining AfriMEDS’ advocacy and CBE goals. A decolonial re-orientation would require re-instating formal, credited instruction and assessment in Sesotho and isiZulu, integrated across all eight competency roles, to close the very communication gaps UFS’s language policy reforms have inadvertently widened.

### COVID-19 as a catalyst for pedagogical transformation

The emergence of COVID-19 as a dominant theme throughout educator interviews, despite its absence from the original research framework, reveals the pandemic’s profound capacity to expose underlying vulnerabilities within the AfriMEDS implementation at UFS. While social distancing protocols necessitated the cancellation of clinical rotations and CBE placements, these disruptions illuminated the precarious nature of a curriculum heavily dependent on face-to-face interactions and traditional clinical settings. The pandemic effectively served as an unintended stress test, demonstrating how external shocks could rapidly dismantle carefully constructed community engagement programmes and interprofessional collaboration initiatives that form the cornerstone of the AfriMEDS framework. Educators reported that “physical interaction is neglected” and that “exposure to patients was diminished this year,” highlighting the curriculum’s insufficient resilience when confronted with systemic disruption. This fragility was particularly evident in CBE components, where the inability to maintain meaningful community partnerships during lockdowns exposed the lack of robust alternative delivery mechanisms. These observations strengthen the argument for developing more adaptable pedagogical models that can maintain their educational integrity across diverse delivery modalities, whether digital, hybrid, or traditional, thereby ensuring that future healthcare professionals acquire essential competencies regardless of external circumstances. The pandemic thus paradoxically accelerated the urgency for the transformative curriculum reform that this research advocates, providing empirical evidence for the necessity of resilient, multi-modal educational approaches within the AfriMEDS implementation framework.

### Institutional accountability and regulatory reform

The web of conflicting priorities identified in this study extend beyond institutional challenges to encompass fundamental deficiencies in regulatory oversight mechanisms that govern medical education in South Africa. The HPCSA, as the statutory body responsible for accrediting medical programmes and ensuring educational quality, bears institutional accountability for the implementation failures documented at UFS. The Council’s 2017 accreditation report highlighted similar concerns regarding CBE and assessment inadequacies, yet no structured follow-up mechanisms emerged in subsequent oversight activities. This pattern suggests that the HPCSA’s current accreditation framework may require fundamental revision to move beyond compliance auditing toward transformative educational support.

What regulatory imperatives emerge from these findings? The evidence presented here demonstrates that existing accreditation standards, while identifying implementation deficits, provide insufficient guidance for meaningful curricular transformation within South African medical schools. As the statutory body responsible for ensuring educational quality and graduate competence, the Council must evolve beyond periodic accreditation visits toward continuous quality improvement partnerships with medical institutions. The regulatory implications of these findings demand immediate attention from the HPCSA, particularly given the documented gaps between policy expectations and institutional capacity for AfriMEDS implementation.

The HPCSA’s next strategic planning cycle (2025–2028) presents a critical opportunity to integrate these research findings into enhanced accreditation frameworks that support rather than merely evaluate AfriMEDS implementation. This transformation requires regulatory mechanisms that facilitate sustainable curriculum reform while addressing the resource constraints and faculty development inadequacies that currently impede competency-based medical education advancement across South African medical schools.

## Conclusion

This evaluation of AfriMEDS implementation at the UFS reveals substantial gaps between policy aspirations and practical reality within South African medical education. The transformative research paradigm effectively illuminated a deep-rooted institutional inertia that traditional evaluation approaches might overlook, demonstrating the value of critical, stakeholder-centred assessment methodologies for curriculum reform initiatives. The developed visual model (Fig. 1) fundamentally transforms how the epistemological foundations of the AfriMEDS physician competency framework can be contextualized within African communities, creating pathways for educational practice that authentically addresses societal healthcare imperatives.

Building directly on the convergent findings from document analysis, educator interviews, and intern surveys, the following phased strategies align with UFS’s specific gaps, best practices identified by the educators and resource realities:

### Strategic recommendations

#### Immediate actions

##### Strengthen faculty development in competency-based assessment

Faculty reported limited capacity to assess non-medical expert roles. A structured faculty development programme, comprising workshops on multisource feedback for collaborator and leadership, plus training in digital portfolio assessment tools, will equip educators to implement reliable, valid evaluations of all AfriMEDS competencies. Integrating patient and community leader perspectives in these engagements will strengthen to transform UFS graduates and educators into catalytic change agents who will drive sustainable healthcare transformation within their communities.

##### Pilot a longitudinal multi-lingual competency programme

Embed structured Sesotho and isiZulu language modules into each CBE rotation will enhance patient rapport in local communities, and prioritize indigenous languages within medical practice (Tervalon & Murray-Garcia, 1998).

##### Standardize reflective portfolio requirements

Implement a unified digital portfolio template across all phases, mandating entries for one community engagement reflection per block. Use portfolios for formative feedback on collaborator and advocacy.

##### Formal professionalism curriculum

Establish a formal professionalism curriculum using structured, case-based ethical dilemmas and guided reflections to move beyond passive observation and ensure all students are explicitly taught professional competencies.

#### Medium-Term reforms (1–3 years) experiences

##### Integrate traditional healing knowledge through community partnerships

Formalize partnerships with accredited traditional healers within CBE modules will create co-taught clinical experiences, directly remedying cultural competency gaps and advancing epistemic decolonisation and pluralism by valuing indigenous knowledge systems (Chitindingu et al., 2014).

#### Transformational goals (3–5 years)

##### Implement ubuntu-centered (person-centered/orientated) assessment frameworks

Educator interviews highlighted that leadership, advocacy, and collaborator competencies remain unassessed in authentic settings. Reframing assessment to include community-health outcome indicators, such as measurable improvements in local COPC projects that aligns evaluation with collective well-being and addresses the observed 37% deficit in leader and manager preparedness.

By sequencing these strategies, starting with capacity building and pilot language modules, progressing to structured interprofessional and leadership experiences, and culminating in full integration of indigenous knowledge and collective community impact metrics. In this way, UFS can transform AfriMEDS implementation from compliance to meaningful community-responsive medical education.

Transformational goals in specific UFS findings can ensure that decolonial pedagogical shifts are both evidence-based and operationally feasible, ultimately producing graduates whose competencies match the needs of South Africa’s diverse communities.

### Regulatory reform and institutional accountability

To address the systemic implementation failures identified in this study, we recommend a comprehensive three-pronged approach to regulatory transformation. First, these research findings should be formally presented to the South African Committee of Medical Deans, the HPCSA Medical and Dental Professions Board during the upcoming accreditation cycle (2025–2027), providing concrete evidence of implementation gaps alongside proposed assessment tools specifically aligned with AfriMEDS competencies. Building upon this evidence base, the HPCSA should develop standardised AfriMEDS assessment guidelines comparable to those established by the Royal College of Physicians and Surgeons of Canada, incorporating mandatory faculty development requirements and benchmarked assessment tools for each competency domain to ensure consistent implementation across institutions.

Finally, to combat the educational isolation reported by UFS educators and foster collaborative innovation, the HPCSA and South African Committee of Medical Deans should establish mandated inter-institutional collaboration mechanisms that enable medical schools to systematically share AfriMEDS implementation strategies and assessment innovations, thereby creating robust communities of practice that can collectively advance the framework’s transformative potential across South African medical education.

### Implications for medical education reform

The study’s findings extend beyond the South African context to offer insights for medical education systems worldwide grappling with competency framework implementation challenges. The demonstrated effectiveness of transformative research paradigms suggests that curriculum evaluation methodologies must evolve to capture power dynamics, cultural contextualisation, and community engagement dimensions that traditional assessment approaches often ignore.

The persistent challenges with non-medical expert competencies, particularly leader, advocacy, and community engagement roles, indicate universal tensions between traditional medical education emphases and contemporary healthcare transformation requirements. How might the extended year at UFS impact the development of these intrinsic competencies? The additional curricular time may provide enhanced opportunities for deeper experiential learning and sustained community engagement, yet it could equally perpetuate existing assessment paradigms that favor traditional medical knowledge over transformative professional capabilities. These findings suggest that successful competency framework implementation requires not merely curriculum modification but fundamental reconceptualization of medical professional identity formation, wherein extended educational duration serves as a catalyst for authentic professional socialization rather than simply prolonged academic preparation.

### Future research directions

Longitudinal studies tracking graduate performance across diverse practice contexts would strengthen understanding of how competency gaps translate to workplace challenges and patient outcomes. Comparative analyses across South African medical schools could illuminate institutional factors that facilitate or hinder successful AfriMEDS implementation, providing evidence-based guidance for curriculum reform initiatives.

Cross-national research examining competency framework adaptation processes could reveal universal principles for culturally responsive medical education reform. Such investigations would particularly benefit resource-constrained environments seeking to balance international accreditation requirements with local contextual needs and community health priorities.

The transformative paradigm’s effectiveness in revealing hidden structural inequities suggests its broader applicability to medical education research. Future investigations should explore how this methodological approach can inform curriculum development, faculty training, and institutional reform processes across diverse educational contexts and cultural settings.

## Data Availability

https://purehost.bath.ac.uk/ws/portalfiles/portal/275718426/N_Mofolo_DBA_Thesis_14_04_2023_final.pdf.
